# Molecular Mechanisms of Recombination Restriction in the Envelope Gene of the Human Immunodeficiency Virus

**DOI:** 10.1371/journal.ppat.1000418

**Published:** 2009-05-08

**Authors:** Etienne Simon-Loriere, Roman Galetto, Meriem Hamoudi, John Archer, Pierre Lefeuvre, Darren P. Martin, David L. Robertson, Matteo Negroni

**Affiliations:** 1 Architecture et Réactivité de l'ARN, Université de Strasbourg, CNRS, IBMC, Strasbourg, France; 2 Institut Pasteur, Paris, France; 3 Faculty of Life Science, University of Manchester, Manchester, United Kingdom; 4 CIRAD, UMR 53 PVBMT CIRAD-Université de la Réunion, La Réunion, France; 5 Institute of Infectious Diseases and Molecular Medicine, University of Cape Town, Observatory, South Africa; The Pennsylvania State University, United States of America

## Abstract

The ability of pathogens to escape the host's immune response is crucial for the establishment of persistent infections and can influence virulence. Recombination has been observed to contribute to this process by generating novel genetic variants. Although distinctive recombination patterns have been described in many viral pathogens, little is known about the influence of biases in the recombination process itself relative to selective forces acting on newly formed recombinants. Understanding these influences is important for determining how recombination contributes to pathogen genome and proteome evolution. Most previous research on recombination-driven protein evolution has focused on relatively simple proteins, usually in the context of directed evolution experiments. Here, we study recombination in the envelope gene of HIV-1 between primary isolates belonging to subtypes that recombine naturally in the HIV/AIDS pandemic. By characterizing the early steps in the generation of recombinants, we provide novel insights into the evolutionary forces that shape recombination patterns within viral populations. Specifically, we show that the combined effects of mechanistic processes that determine the locations of recombination breakpoints across the HIV-1 envelope gene, and purifying selection acting against dysfunctional recombinants, can explain almost the entire distribution of breakpoints found within this gene in nature. These constraints account for the surprising paucity of recombination breakpoints found in infected individuals within this highly variable gene. Thus, the apparent randomness of HIV evolution via recombination may in fact be relatively more predictable than anticipated. In addition, the dominance of purifying selection in localized areas of the HIV genome defines regions where functional constraints on recombinants appear particularly strong, pointing to vulnerable aspects of HIV biology.

## Author Summary

Recombination allows mixing portions of genomes of different origins, generating chimeric genes and genomes. With respect to the random generation of new mutations, it can lead to the simultaneous insertion of several substitutions, introducing more drastic changes in the genome. Furthermore, recombination is expected to yield a higher proportion of functional products since it combines variants that already exist in the population and that are therefore compatible with the survival of the organism. However, when recombination involves genetically distant strains, it can be constrained by the necessity to retain the functionality of the resulting products. In pathogens, which are subjected to strong selective pressures, recombination is particularly important, and several viruses, such as the human immunodeficiency virus (HIV), readily recombine. Here, we demonstrate the existence of preferential regions for recombination in the HIV-1 envelope gene when crossing sequences representative of strains observed to recombine in vivo. Furthermore, some recombinants give a decreased proportion of functional products. When considering these factors, one can retrace the history of most natural HIV recombinants. Recombination in HIV appears not so unpredictable, therefore, and the existence of recombinants that frequently generate nonfunctional products highlights previously unappreciated limits of the genetic flexibility of HIV.

## Introduction

Pathogens, and viruses in particular, are subject to strong selective pressures during infection and often have characteristically high degrees of genetic variation [1]. Recombination is an important evolutionary mechanism that contributes to this genetic diversification. By creating novel combinations of pre-existing genetic polymorphisms in a single replication cycle, recombination enables greater movements through sequence space than can be achieved by individual point mutations. As a consequence, recombination provides access to evolutionary “shortcuts”. In addition, since recombination generally involves genes that already encode functional products, the probability of producing viable progeny is higher compared to the insertion of an equivalent number of random point mutations [2]. However, the generation of recombinant forms is not an unconstrained process. Genes and genomes generally evolve through the slow accumulation of point mutations, which often requires the progressive insertion of compensatory mutations at “linked” sites. This coevolution permits the preservation of epistatic interactions. By simultaneously introducing several substitutions, recombination has the potential to substantially perturb such coevolved intra-genome interaction networks [2],[3], impairing the functionality of the genes involved. Thus, the balance between the advantages of taking evolutionary shortcuts and the risk of chimeras being dysfunctional [2] determines the role played by recombination in the evolution of a given gene or organism.

Several studies have focused on the impact of recombination on the evolution of proteins, particularly in relation to directed evolution experiments [4],[5]. Two major factors have a large influence on the functionality of recombinants proteins. The first is the position of recombination breakpoint (the region where the sequence shifts from that of one parental sequence to the other) relative to the location of genetic polymorphisms within the gene. Recombinants involving a large number of non-synonymous substitutions will in fact have a low probability of being functional [2]. The second factor is the position of the breakpoints in relation to the boundaries of discrete protein folds. Breakpoints near the boundaries of these domains will in general have a smaller impact on protein folding, and hence protein function, than breakpoints occurring within them [3],[6],[7]. Recent work on Begomoviruses corroborated these findings by demonstrating that recombination events found in natural viral populations are significantly less disruptive of protein folding than randomly generated recombinants [8].

Adaptation of pathogens, either to on-going immune pressures within individual hosts or following transmission to new hosts of the same or different species, can result in infectious outbreaks that constitute major threats for public health [9]–[12]. The human immunodeficiency virus (HIV) is an extremely recombinogenic pathogen in which recombination has been implicated in key aspects of viral pathogenesis such as immune evasion [13], transmissibility [14], the evolution of antiretroviral resistance [15],[16] and cross-species transmission [9],[12]. Indeed, the remarkable genetic flexibility of HIV is underlined by its large genetic diversity. The HIV-1 population is subdivided into three groups, named M, N and O, with group M (which is responsible for the vast majority of the infections worldwide) being further subdivided into nine subtypes (named A, B, C, D, F, G, H, J and K) [17].

Although recombination in HIV has been shown to occur at all phylogenetic levels (intra- and inter-subtype, as well as inter-group, reviewed in reference [18]), the most widely noted impact of recombination on the genetic diversification of this virus is the frequent natural occurrence of inter-subtype recombinants in parts of the world where multiple subtypes co-circulate [19]–[22]. When the same inter-subtype recombinant is transmitted between multiple individuals, and has therefore the potential to be of epidemiological significance, it is termed a Circulating Recombinant Form (CRF) [17]. As with the HIV-1 subtypes, CRFs form distinct clusters in phylogenetic trees and some of them contribute substantially to the pandemic.

Sufficient inter-subtype recombinant sequences have been sampled to permit the detailed characterisation of variation in the locations of breakpoints both within individual genes [22],[23], and entire genomes [24],[25],[32]. This makes HIV a particularly useful model for studying the forces that shape pathogen populations within the context of global epidemics. Here we focus on recombination within the envelope gene (*env*). This gene encodes two polypeptides (gp120 and gp41) that form a heterodimer at the surface of the viral particle. Trimers of these heterodimers are the functional units that are responsible for binding to the cellular receptors and co-receptors and ultimately lead to viral entry into target cells [26]. The two protein products of *env* are also the targets of all the neutralising antibodies identified to date [27]. By using a tissue culture system to characterise inter-subtype recombinants generated within *env* in the absence of selection, and assaying the functionality of recombinant genes, we produce an empirical model of HIV recombination that accurately describes recombination patterns found in viruses sampled throughout the HIV pandemic.

## Results

### Recombination pattern in the *env* gene

We used different combinations of *env* sequences from primary HIV-1 isolates belonging to either different group M subtypes or group O (see Materials and Methods for the list of parental isolates used) to determine the distribution of breakpoints occurring within the HIV *env* gene in the absence of selection. We chose combinations of isolates belonging to subtypes that are co-circulating in regions of the world from which natural inter-subtype recombinant forms have emerged [28].

**Figure 1:**
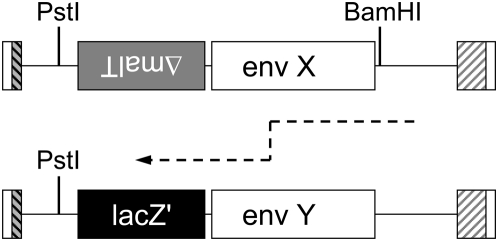
Recombination during a single cycle of infection of cells in culture. Schematic representation of the structure of the genomic RNAs used for the recombination assay. White box at both ends of the RNAs: R sequence; grey–black hatched box: U5 sequence; white–grey hatched box: partially deleted and therefore non-functional U3 sequence; white boxes: HIV envelope sequences studied, *env X* and *env Y* stand for sequences of two different isolates (isolate X on the donor RNA and isolate Y on the acceptor, respectively); black box: marker *lacZ'* bacterial gene; grey box: partial sequence of the bacterial gene *malT*, inserted in the reverse orientation. The approximate location of the BamHI site (present only on the donor RNA) and of the PstI site present on both RNAs is also indicated. The path followed by reverse transcription for generating the BamHI^+^/LacZ'^+^ recombinants studied in the present work is indicated schematically.

In order to quantify variations in recombination rates across *env* we used a previously described experimental system where human T cells are transduced with HIV-1 replication-defective vectors pseudotyped with the Vesicular Stomatitis Virus (VSV) envelope [29]. As this system mimics a single cycle of viral infection in which reverse transcription products neither influence cellular survival, nor confer a specific phenotype to the transduced cells, recombinants that were produced during reverse transcription were not subjected to any selection. After cloning of the reverse transcription products in *E. coli*, the system enabled identification of the recombinants based on the presence of a *lacZ* reporter gene (Figure 1). Given that known input sequences were used, such an approach enables the accurate and unambiguous localization of the breakpoint position to precise regions bounded by nucleotides that differ between the two parental sequences.

**Figure 2:**
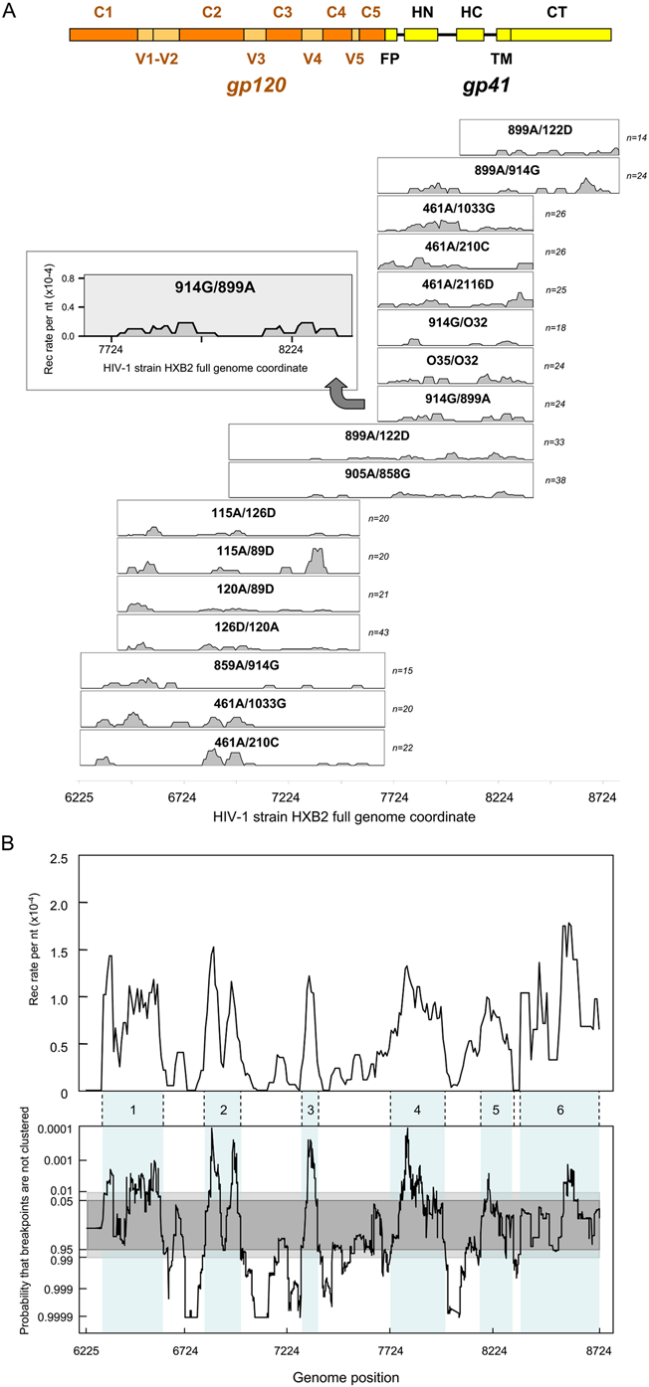
Recombination breakpoint distributions determined in a selection-free experimental setting. (A) Recombination pattern along the *env* gene with the different pairs of isolates studied. Recombination rates per nucleotide observed after a single infection cycle are plotted as a function of the position along the *env* gene, as indicated in the example of the 914G/899A pair, given in the insert (the identity of the parental isolates is given as donor/acceptor). Nucleotide positions are given, throughout the article, in relation to the position on the HXB2 isolate genome. Recombinants 115A/126D, 115A/89D, 120A/89D, and 126D/120A were described previously [33]. The number (*n*) of individual recombinants for which the position of the breakpoint has been mapped is given on the right of each graph. A map of gp120 and gp41 domains is given as a frame of reference at the top of the figure. (B) top graph: pooled distribution of recombination breakpoints across *env*, obtained as described in Materials and Methods. Bottom graph: the height of the black plot at any particular position represents the probability (determined by a permutation test with 10,000 iterations) that recombination breakpoint distributions are not more clustered than would be expected by chance within a 50 nucleotides window centred on that position. Assuming that breakpoints are randomly distributed, the dark and light grey regions represent degrees of breakpoint clustering expected due to chance in 95% and 99% of the examined windows, respectively. Whereas peaks emerging above the grey regions represent possible recombination hot-spots, troughs dipping below the grey regions represent possible recombination cold-spots. The pale-blue shaded areas numbered from 1 to 6 correspond to breakpoint clusters, or hot regions, as defined in the text.

The regions of the envelope gene that were studied were chosen so as to obtain 700 to 1,500 nucleotides overlapping windows, spanning the whole of *env*. For each of seventeen different combinations of parental sequence pairs (Figure 2A), a recombination rate per nucleotide and per reverse transcription run was calculated within a 50 nucleotides sliding window (with 10 nucleotides step size). These were plotted as a function of the location of the window along the gene. To evaluate whether recombination-prone regions exist within the population, data from the 17 different pairs of parental sequences were pooled and an average recombination rate was computed for the different regions, and plotted as function of the position along the *env* gene (Figure 2B, top panel). Peaks and troughs were apparent all along the gene, with regions refractory to recombination being more common in the gp120 coding portion than in the gp41 region. The probability that breakpoints were more or less clustered across *env* than could be accounted for by chance (given the null hypothesis that breakpoint positions occur randomly) was determined by a permutation test (Figure 2B, bottom panel). Six major recombination-prone or “hot” regions (shaded light blue areas in Figure 2B) could be defined as *env* regions where breakpoint clusters were bounded by statistically significant breakpoint “cold spot” (p<0.05). Each of the six identified breakpoint clusters contained at least one breakpoint cluster that constituted a statistically significant recombination “hot-spot” (p<0.01). While these recombination-prone regions covered only slightly more than half of the whole gene (55.3%), they included 81.6% of all the breakpoints (337/413) mapped. These six hot regions are areas where recombination occurs preferentially during HIV replication, irrespective of the parental strains involved.

### Selection for functional recombinants

We next investigated the fate of these recombinants with respect to their establishment in the natural HIV-1 population. The fixation of a recombinant gene within a population is dependent on the interplay of multiple factors. Nevertheless, an obligatory component of evolution is undoubtedly the elimination by purifying selection of viruses that express dysfunctional proteins. To evaluate how profoundly this aspect of natural selection might influence the pattern of breakpoints generated by the mechanism of recombination, we determined the relative functionality of a subset of recombinant *env* genes.

In addition to encoding the proteins that coat the viral membrane, *env* also encodes a well-known functional RNA structure, the Rev responsive element (RRE). For the recombinants containing breakpoints in the RRE region the functionality of this RNA module was therefore also tested. Being involved in the regulation of the timing and the balance among the various forms of unspliced and partially or completely spliced RNAs, RRE is essential for viral replication [30]. Failure to properly regulate this process results in either a decrease or complete halt in viral production [30]. The functionality of chimaeric RREs was tested by measuring viral titres obtained upon transfection of cells with a plasmid containing the proviral sequence of the molecular clone NL4.3 of HIV-1, in which we had replaced the native NL4.3 RRE with that of the various chimaeric RREs. To uncouple the effects on RNA-folding caused by the introduced RRE sequences from those altering the amino acid sequence of expressed proteins, we used a variant of NL4.3 that does not express Env (NL4.3-Env^−^) [31], and a plasmid encoding the wild-type Env was co-transfected to complement the production of gp120 and gp41 proteins. In order to increase the statistical power of the analysis, additional chimaeric RREs were constructed using parental sequences other than those employed in our cell culture recombinant generation experiments (following a PCR procedure described in Materials and Methods) and tested for their functionality. As can be seen in Table 1, the viral titres obtained with every chimaeric RRE sequence we tested were both similar to those obtained with non-recombinant parental RRE sequences and markedly higher than that observed when the RRE was replaced with a non-viral sequence (see Materials and Methods). This result therefore clearly indicated that recombinants generated by breakpoints within the RRE generally retain the functionality of this element.

**Table 1:** Functionality test of recombinant RRE structures.

Sample Name	pg/µl of p24 Antigen	Standard Deviation
RRE off ΔdNK	17	12
Parental A905	346	14
Parental G914	389	26
Parental O32	296	18
AG 1647	388	73
GA 1707	317	35
GA 1892	325	16
AG 1757	267	36
AG 1657	367	32
GA 1637	245	53
AG 1757	288	33
AC 7899	338	33
AC 7987	325	27
GB 7935	283	15
GC 7935	301	43
OG 7810	318	12
OG 8090	234	45

To determine the functionality of individual recombinant envelopes at the protein level, full-length recombinant envelope genes containing breakpoints of interest were constructed by successive PCR, as described in Material and Methods. Each full-length recombinant gene was then cloned in the pcDNA3.1 expression vector, and used to transfect HEK 293T cells together with the pNL4.3-Env^−^-Luc plasmid, to generate viral particles pseudotyped with the recombinant envelope of interest. The functionality of the recombinant envelopes was then tested after transduction of HEK 293T-CD4^+^-CCR5^+^ cells at a multiplicity of infection of 0.1, by measuring luciferase expression in these cells 48 hours after transduction. Since target cells cannot synthesize new viral envelope proteins, infection was limited to reverse transcription and, potentially, integration. The luciferase values observed therefore reflected the relative success of viral entry into the target cells. For this analysis recombinants derived from parental *env* sequences that yielded the strongest positive signals in this single cycle test were chosen (parental sequences A-Q461, C-CAP210, G-1033 and O-32, see Table 2 for their relative genetic distance) due to the higher reliability of the luciferase signal. The parental *env* sequences were used as controls. As for the functional analysis of the RRE, additional recombinants involving combinations of parental sequences – other than those involved in the experiments of recombination in cell culture, but carrying breakpoints in the same regions – were also tested. These additional recombinant *env* sequences were generated by PCR, as described for the reconstitution of the full-length *env* gene.

**Table 2:** Identity between different pairs of parental sequences.

	A-Q461 (2598 nt)	B-THRO (2622 nt)	C-CAP210 (2595 nt)	G-1033 (2577 nt)	O-32 (2619 nt)	HXB2 (2568 nt)
A-Q461	—	0.721	0.744	0.734	0.510	0.718
B-THRO	**0.782**	—	0.710	0.698	0.491	0.798
C-CAP210	**0.796**	**0.772**	—	0.726	0.501	0.711
G-1033	**0.800**	**0.77**	**0.787**	—	0.507	0.713
O-32	**0.581**	**0.564**	**0.578**	**0.581**	—	0.486
HXB2	**0.792**	**0.870**	**0.784**	**0.790**	**0.574**	—

Regular text gives the level of identity at the nucleotide level, bold text at the amino acid level.

**Figure 3:**
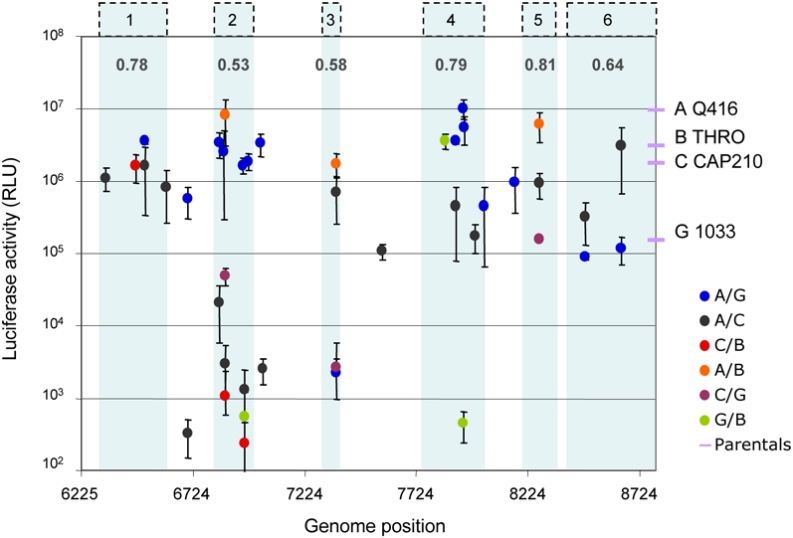
Functionality of recombinant envelope proteins. Functionality of the recombinant proteins as a function of the breakpoint position (in nucleotides) along the *env* gene. Each individual recombinant tested is indicated by a circle. Error bars indicate standard deviations observed in four independent experiments. The luciferase values that were determined for the four parental strains used to generate the recombinants are represented, as a frame of reference, by lilac bars on the right. The six recombination-prone regions defined in Figure 2B are shaded in pale blue and annotated accordingly above the graph. The value of loss of functionality approximated for each region is given in bold in the top part of the graph. Three M/O inter-type recombinants were also tested (AO456, AO7810, and AO8090, breakpoint positions 6508, 7810, and 8090, respectively, of the HXB2 reference strain), resulting in a complete loss of functionality, probably due to the higher sequence divergence between the parental isolates. In order to preserve the homogeneity of the dataset to intersubtype recombinants, these recombinants are neither presented in the figure, nor were considered for the calculation of the average functionality of the recombinants, described in the main text.

Luciferase values determined for each recombinant were plotted as a function of the corresponding breakpoint position (Figure 3). Recombinants with breakpoints falling within the six hot regions indicated in Figure 2B were preferentially characterized. It was apparent that most of the severely defective recombinants contained breakpoints in hot regions 2 and 3 of the recombination rate distribution (Figure 3). Given this data, we approximated a probability of Env functionality being disrupted by breakpoints falling within each of the six high recombination-rate regions. Since the parental sequences themselves were not uniformly functional (Figure 3), a situation that is probably common in nature, for each recombinant an estimate of loss of functionality was calculated by dividing the luciferase value obtained with that recombinant by the one of the least functional parental sequence involved in its generation. Recombinants displaying values between those of the two parental sequences were considered to retain functionality (and assigned a functionality value of 1). Of note, none of the recombinants yielded functionality values higher than that of the most functional parent from which it was generated. Values from recombinants containing breakpoints within the same region of the six hot regions were pooled, and a functionality loss value for each region was averaged (Figure 3). The most significant loss of functionality was observed in regions 2, 3, and 6.

### Natural recombination breakpoint distributions essentially mirror those of the functional recombinants generated in tissue culture

**Figure 4:**
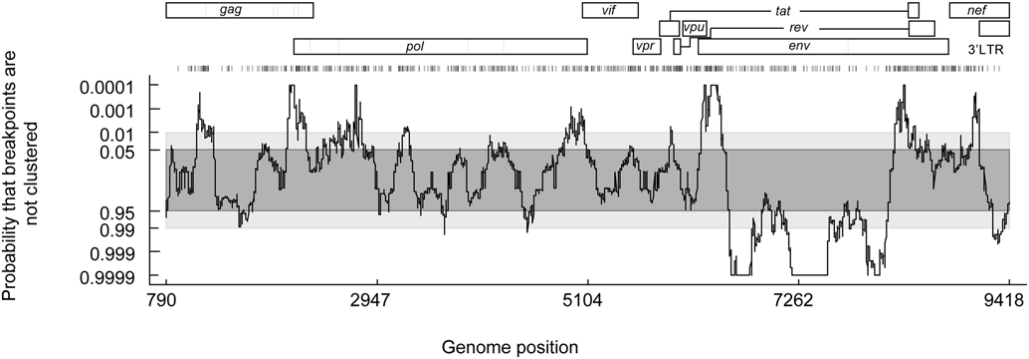
Natural recombination patterns across the HIV genome. The representation is the same as for the bottom panel of Figure 2B. The row of vertical lines above the plot represents the inferred positions of the 691 breakpoints used in its construction. The genome map of the HXB2 HIV-1 group M strain is given as a frame of reference.

Having defined a pattern of recombination in the absence of selection and the approximate probabilities of recombination events in various parts of *env* yielding fully functional products, we were interested in determining whether our experimental data could explain breakpoint patterns observed in circulating recombinants. The distribution along the whole HIV genome of 691 recombination breakpoints within HIV-1 group M full genome sequences from the LANL HIV Sequence Databases (http://hiv-web.lanl.gov/) was inferred. The same approach used in Figure 2B to define the probability that at any region of the genome the breakpoints were more clustered than would be expected by chance was used, with a 200 nucleotides window. A previous analysis of HIV recombinants modelled the distribution of breakpoints and indicated a significant clustering of breakpoints in the 5′ and 3′ ends of the envelope gene and a lack of breakpoints between these regions [32]. Our new analysis (Figure 4) confirmed the propensity for breakpoints to be located at the 5′ and 3′ ends of the *env* gene and the lack of breakpoints in the majority of its internal regions in recombinants from the database.

**Figure 5:**
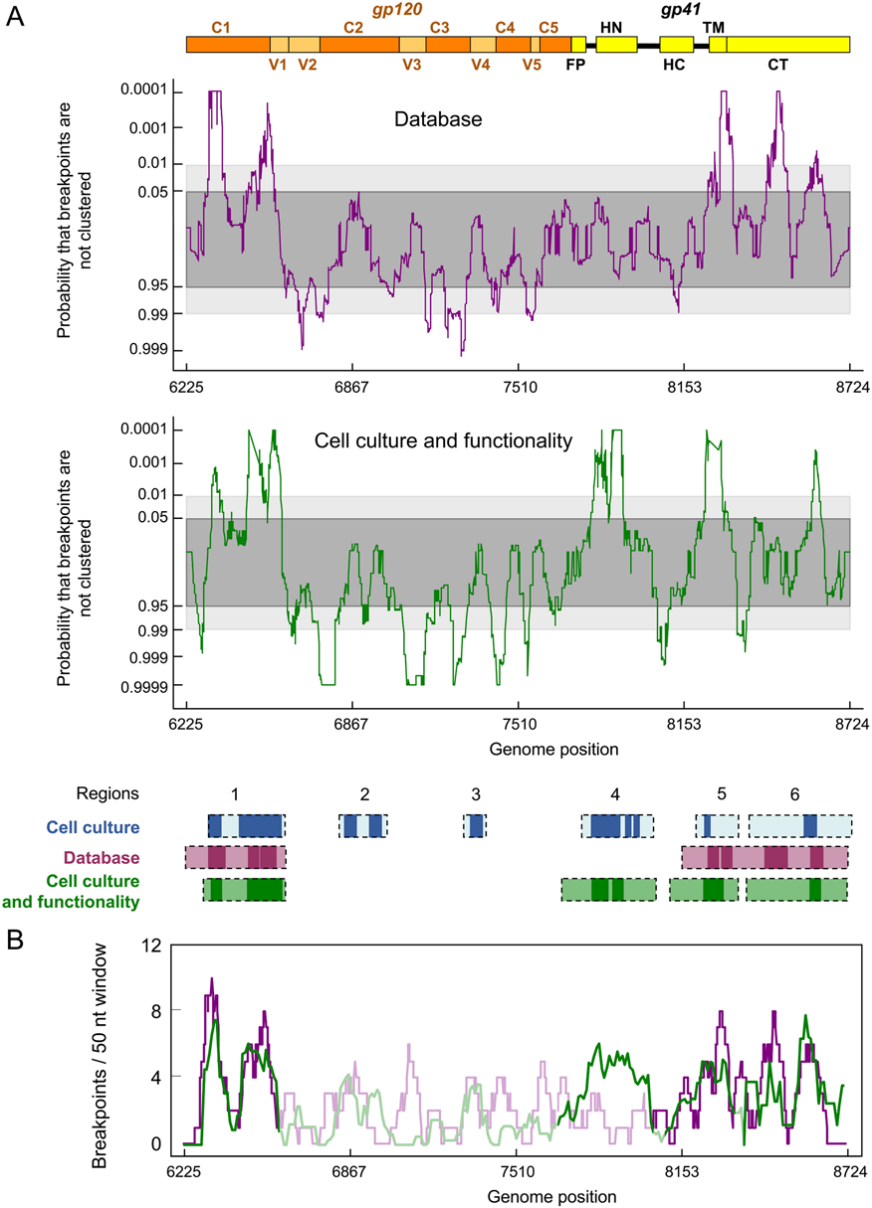
Natural recombination breakpoint distributions can be essentially accounted for by a combination of mechanistic recombination rate variations and selection against dysfunctional recombinants. (A) The recombination breakpoint distribution detectable using HIV 1 group M *env* genes sampled from nature (purple) and in cell culture after correction for the functionality of the recombinant products (green). The representations are the same as for the bottom panel of Figure 2B. Boxes at the bottom of the figure define the hot recombination regions (as defined in the text) identified in cell culture, amongst natural HIV recombinants, and in cell culture after correction for the functionality of the recombinants. Darker boxes indicate statistically significant hot-spots where breakpoints are particularly tightly clustered (p<0.01). A map of gp120 and gp41 domains is given as a frame of reference at the top of the panel. (B) The purple plot represents the distribution of breakpoints observed in natural HIV recombinants and the green plot the mean expected distribution based on our empirical model formulated using “functionality correction” of our breakpoint distributions determined in cell culture. Regions outside the recombination hot regions shown by the boxes in the lower portion of (A) of the figure are shaded.

In order to compare our experimentally determined breakpoint distribution to that found in recombinants from the HIV Sequence database, a higher-resolution view of the breakpoint distribution within the *env* gene was determined using the positions of 133 unambiguously unique recombination breakpoints detectable within 230 *env* sequences. Following the same procedure described above, but using a 50 nucleotides window to enable detection of breakpoint clusters at the same resolution as in our experimental system, we identified a series of recombination hot- and cold-regions within the gene (Figure 5A, purple graph). In a similar way to the breakpoint distribution detected in cell culture, various hot regions could be defined (light-purple boxes at the bottom of Figure 5A), which corresponded remarkably well to recombination hot regions 1, 5 and 6 seen in cell culture (light-blue boxes). Whereas the other hot regions identified in cell culture had no corresponding counterparts in the natural breakpoint distribution, there was close correspondence between the cold-spots detected in both distributions.

Next we used the SCHEMA-based method [8] to investigate whether or not this breakpoint distribution exhibits evidence of purifying selection acting on recombinants with disrupted protein folding. This analysis indicated that breakpoints observable in natural viruses tend to occur in regions within *env* that were predicted to have a significantly lower impact on protein folding than randomly placed breakpoints (p<1.0×10^−4^ for gp120 and p = 8.9×10^−3^ for gp41, see Protocol S1). To investigate whether accounting for variations in the functionality of recombinants might reconcile the natural and experimental breakpoint distributions, we first approximated the combined effects of mechanistic recombination rate variation (Figure 2B) and selection for fully functional recombinants (Figure 3) on the distribution of breakpoints in cell culture. Selection “corrected” recombination rate estimates were then used to determine the distribution of 133 expected breakpoints. The resulting distribution was used to evaluate the probability of clustering of breakpoints (green graph in Figure 5A). Only regions 1, 4, 5 and 6 remained areas of significant clustering (light-green boxes at the bottom of Figure 5A), a pattern very close to that found in HIV recombinants sampled from nature, with the exception of region 4 for which there was substantially less evidence of recombination within natural recombinants than was expected based on our empirical model. Indeed, when compared to the distribution found for the 133 breakpoints encountered in the natural HIV recombinants (Figure 5B), a remarkable overlap was observed, with the discrete statistically significant breakpoint clusters being consistently recaptured by our empirical model of env recombination. The substantial difference of recombination rates in region 4 was also clear.

## Discussion

Through the functional characterization of HIV envelope genes generated by recombination in the absence of selection, we retrace the early steps shaping patterns of inter-subtype *env* recombination found in the HIV-1 pandemic. We observe that the mechanism of recombination alone defines regions where recombination occurs at significantly higher rates than elsewhere along the gene. The existence of such regions is strongly suggestive of spatially conserved features in HIV genomes that either promote or restrict recombination between different isolates. The distribution of breakpoints within the gp120 encoding region (Figure 2B) is likely due to the distribution of conserved and variable regions, the latter restricting recombination because of the low degree of local sequence identity between the parental sequences [32],[33].

Within genomic regions where sequence identity is high, a trigger for recombination could be the presence of secondary structures [34]. The highest recombination peak within the second region in Figure 2B (corresponding with the C2 portion of gp120) coincides with a recombination hot-spot that is determined by the presence of a stable RNA hairpin structure [29],[35],[36], while the fourth hot region (Figure 2B) corresponds to the RRE RNA structure that is highly conserved amongst all HIV isolates [37]. It is therefore possible that RNA secondary structures also contribute to the high rates of recombination observed at some of the other recombination hot regions. Noteworthy, the functionality of the RRE was retained even when crossing genetically distant isolates as for inter-group M/O recombinants (Figure 2A), supporting the possibility that regions of the genome harbouring functional RNA structures, which are generally more conserved within the population, provide a mechanism for crossing distantly related retroviruses and are possibly important for recombination of RNA viruses in general.

With respect to selection of recombinant genes at the protein level, experiments involving lattice proteins have shown that genes encoding proteins that tolerate mutations also tend to be recombination tolerant [2]. Since the *env* gene displays a degree of diversity between isolates from different HIV-1 group M subtypes ([38] and references herein) that is two to three times higher than the genome average, we anticipated that the manifest mutation tolerance of *env* might predispose it to high recombination tolerance. However, we show that this is not the case with certain regions within the gp120 encoding portions of *env* (particularly region 2 described in the present work in Figure 3) tending not to tolerate recombination well.

Viruses with small genomes (including all RNA viruses) tend to use overlapping genes expressed in different reading frames and to encode proteins that have multiple functions. The HIV envelope encodes for such proteins [26], and the subtle biochemical equilibrium that regulates their functionality is very possibly limiting tolerance to recombination. The low recombination tolerance of the gp120-encoding region could only be imprecisely predicted based on computational estimates of recombination induced protein fold disruption using the SCHEMA algorithm [3]. This may have been due to either our SCHEMA analyses being based on incomplete gp41 and gp120 structures or the fact that the structures used only reflected a single conformation of these two proteins. Therefore this analysis neither takes into account the conformational changes required for Env functionality, nor the quaternary arrangement of the proteins within Env trimers. Despite these issues, the SCHEMA analysis indicated that, amongst the HIV *env* sequences sampled from nature, selection has been acting against recombinants with disrupted protein folding (Table S1). Unravelling the molecular reasons for the reduced functionality of certain recombinants could provide valuable insights into the nature of the molecular interaction networks required for proper Env function.

The specific determinants of viral fitness (or *in vivo* replicative capacity) are complex and poorly understood at present. The fixation of a recombinant gene within a population is likely to depend on the interplay of multiple factors. Although combining cell culture functionality data with recombination rate heterogeneity is an oversimplified view of this process, the pattern of recombination predicted by our empirical model matches remarkably well the breakpoint distributions observed in nature (Figure 5B). The only major deviation from this was constituted by the fourth recombination hot region observed in cell culture, which was absent from the natural breakpoint distribution (Figures 2B and 5B). Determining the reasons for this discrepancy will improve our understanding of the mechanisms governing the success of recombinants in nature.

Although the host immune response certainly plays a significant role in the selection of recombinant variants *in vivo* [13], the similarities between the natural and experimental breakpoint distributions suggest that the forces responsible for the selection of recombinants *in vivo* only have limited impact on inter-subtype breakpoint patterns in *env*. This is most likely due to a combination of factors including mainly the complex epistatic interactions within *env*, the high density of fitness-determining loci within this gene, and the biochemical mechanism of recombination, which collectively constrain the fixation of genetic variability introduced by recombination. Negative fitness effects associated with recombination in *env*, however, should decrease with decreasing parental genetic distances [3],[6],[39] and therefore, in the context of intra-subtype recombination, the selective constraints on recombinants should be more relaxed than we have found them to be here.

Considering recombination in *env* in the context of the rest of the HIV genome, it is apparent that *env* displays the most dramatically variable natural breakpoint distribution of all HIV genes [24],[32], and it constitutes the only gene within which there is an extended region with limited recombination (Figure 4). Nevertheless, although less marked, breakpoint distribution patterns reminiscent of those found in *env*, with alternate clusters and troughs are also identifiable in several other regions of the genome such as *gag* and *pol* [32] (Figure 4). Although little information is presently available either on differential mechanistic predispositions to recombination across these regions, or on the functionality of the resulting products, it is tempting to speculate that underlying rules such as we have defined here for *env* may also be operational in these other cases.

In conclusion, by experimentally reproducing the generation of HIV-1 recombinants, we demonstrate that the distinctive distribution of breakpoints found in natural viruses is strongly shaped by both the mechanism of recombination, and the relative functionality of the recombinant genes. Thus, HIV evolution might not be the relentlessly unpredictable process it sometimes seems, and exploiting this evidence to pre-empt and counter the most favoured evolutionary tactics of this virus may ultimately be an efficient means by which we can devise effective vaccines and improve drugs against the virus.

## Materials and Methods

### Cell culture

HEK 293T, and CD4^+^CCR5^+^ 293T cells were grown in Dulbecco's modified Eagle's medium supplemented with 10% foetal calf serum, penicillin, and streptomycin (from Invitrogen, CA, USA), and maintained at 37°C with 10% CO_2_. MT4 cells were maintained in RPMI 1640 medium supplemented with 10% foetal calf serum and antibiotics at 37°C with 5% CO_2_.

### Viral sequences

The parental isolates used in this study were A-115, A-120, A-899 [33], A-859, A-905 (from S. Saragosti) and A-Q461 (Gene Bank: AF407156) for subtype A isolates; B-THRO (Gene Bank: AY835448), for subtype B; D-126, D-89, D-122, [33] and D-21.16 (Gene Bank: U27399), for subtype D; C-CAP210 (Gene Bank: DQ435683), for subtype C; G-858, G-914, (from S. Saragosti) and G-MP1033 (from M. Peeters, Gene Bank: AM279365), for subtype G; O-35 and O-32, for group O (from S. Saragosti).

### Single cycle tissue culture recombination assay

Single cycle recombination assays were performed using a system previously developed by our laboratory [29]. HIV-1 *env* fragments from group M subtypes A, C, D and G, and from group O viral DNA were amplified by PCR from infected PBMCs obtained from patients and cloned in plasmids (called genomic plasmids), which differ for the genetic marker present downstream (in the sense of reverse transcription) of the sequence in which recombination is studied (Figure 1). All constructs were verified by sequencing. The trans-complementation plasmids, pCMV R8.2 [40] encoding HIV-1 Gag, Pol, and accessory proteins, and pHCMV-G [41] encoding the Vesicular Stomatitis Virus envelope protein were co-transfected into 293T cells with the two genomic plasmids to produce defective retrovirus particles which were then used to transduce MT4 cells as previously described [29]. The reverse transcription products were purified from the cytoplasmic fraction of transduced cells using the method described by Hirt [42]. The purified double stranded DNA was digested with DpnI for 2 h at 37°C (in order to eliminate possible contaminating DNA of bacterial origin) prior to PCR amplification as previously described [29]. The amplified product was purified after electrophoresis on agarose gel, digested with PstI and BamHI, ligated to an appropriate plasmid vector and used to transform *E. coli*. Plating on IPTG/X-Gal containing agar plates allowed blue/white screening of recombinant and parental colonies, respectively [29]. The frequency of recombination was determined by computing the number of blue colonies over the total number of colonies as described in reference [29]. Recombination breakpoints were identified by full-length sequencing of the *env* portion of the recombinant clones.

### Analysis of recombinants generated after a single infectious cycle in tissue culture

The recombinant and parental sequences of each pair of isolates tested were aligned using CLUSTAL X [43]. The breakpoint location of each recombinant was determined as being the central position of the interval bounded by the two closest nucleotide sites that were characteristic of each of the parental sequences). Recombination rates were calculated as follows. We define each recombination window studied with each pair of parental sequences as *RwXY_a–b_*, for a recombination window involving isolates *X* and *Y*, spanning position *a* to position *b* of *env* (reference sequence HXB2); a 50 nucleotides window was then considered (*XY_a–b_Sw_i_*, for a sliding window starting at position *i* of *env*), beginning from the 5′ border of the sequence studied and the number of breakpoints (indicated as *XY_a–b_n_i_*) falling within the window was counted. The resulting recombination rate per nucleotide in the sliding window *XY_a–b_Sw_i_* is
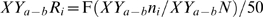
where *XY_a–b_N* is the total number of breakpoints characterized for the *RWXY_a–b_* pair, and 50 is the size in nucleotides of the sliding window, and F the frequency of recombination observed in the whole region studied, as defined in the previous chapter. The sliding window was then displaced with a 10 nucleotides increment (resulting in *XY_a–b_Sw_i+10_*, *XY_a–b_Sw_i+20_*, … ) across the recombination window, and *XY_a–b_R_i+10_*, *XY_a–b_R_i+20_*, … were computed. The various R values were reported in the graph as a function of the position of the midpoint of the window along the gene (i.e. the position of the 25^th^ nucleotide of each sliding window). For the pooled dataset reported in Figure 2B, the analysis based on the sliding window was repeated. If *Swp_i_* stands for the sliding window at position *i* for the pooled dataset, *Rp_i_* for the corresponding recombination rate, and *q* is the number of recombination window including position *i*, recombination rate at position *i* is calculated as
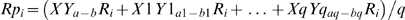
To statistically test for the presence of recombination hot and cold-spots in the experimentally determined recombination breakpoint distributions we used a modification of a permutation test described previously [44]. Unlike in analyses of natural recombinants, the breakpoint positions approximated in our experimental procedure were not subject to biases introduced by underlying degrees of parental sequence nucleotide variability and patchiness of parental sequence sampling. Rather than explicitly accounting for these biases when placing randomised recombination breakpoints as in the permutation test described by Heath et al. [44], our modification of the test involved the completely randomised placement of recombination breakpoints. The test essentially involved the randomised recreation of 10,000 versions of our real dataset with each version containing exactly the same number of breakpoints between the same 17 parental sequence pairs observed in the real dataset. From breakpoint distributions determined for each of these 10,000 randomised datasets we were able to work out confidence intervals for expected breakpoint density variation given the completely random occurrence of recombination.

For simulating the distribution of 133 breakpoints based on the combined effects of (i) the mechanistic recombination rate and (ii) selection for functional recombinants, local recombination rate data used to generate the graph in Figure 2B were first multiplied by the respective functionality scores given in Figure 3 for each corresponding region, yielding “functionality corrected” rates for each region. Once the expected breakpoint distribution of 133 unique recombinants determined by this method, the number of breakpoints present in a 50 nucleotides rolling window, sliding with a 10 nucleotides increment was calculated and plotted (in Figure 5B) as function of the position along the gene. Deviations from expected degrees of breakpoint clustering given the null hypothesis of random breakpoint locations, was tested using the same modification of the Heath et al., [44] permutation test detailed above.

### Construction of full-length recombinant envelope genes, and recombinant RREs by PCR

Full-length sequences of recombinant *env* genes were reconstituted, using an overlapping PCR procedure. We separately amplified the region from the 5′ end of the acceptor gene (using primer Topo5′ annealing to positions 5966–5990 of the reference strain HXB2) to the breakpoint position (using a specific primer encompassing the region of the breakpoint) and from the 3′ end of the donor gene (primer Donor3′, HXB2 positions 8785–8819) to the breakpoint position (also in this case with a specific primer). These PCR products, overlapping by approximately 30 nucleotides around the breakpoint site, were mixed at equal ratios and used as templates to generate the full-length recombinant *env* gene using primer Topo5′ and Donor3′. All PCR reactions were run with Phusion DNA polymerase (Finnzymes, Finland) for 30 cycles. PCR products were gel purified and ligated to pCDNA3.1 Topo (Invitrogen, CA, USA). For RRE functionality assays, a portion of the envelope gene containing the RRE of pNL4.3-Env^−^-Luc (nucleotides 7646 to 8046) was replaced with the corresponding sequence of parental or recombinant envelope genes or, as a negative control, a 400 nt sequence from the *Drosophila melanogaster* desoxynucleoside kinase gene (ΔdNK). All constructs were verified by sequencing.

### Functionality of RRE sequences

HIV particles were produced by co-transfection of HEK 293T cells with an expression vector for a CCR5-tropic (ADA) HIV-1 envelope [45] kind gift of Dr. M. Alizon, together with a pNL4.3-Env^−^-Luc containing either a parental or recombinant RRE sequence or ΔdNK. Forty-eight hours post transfection, supernatants were filtered trough a 0.45 µM filter and p24 levels were determined using the HIV-1 p24 enzyme-linked immunoabsorbent assay kit (PerkinElmer Life Sciences, MA, USA).

### Functionality of Env proteins

Reporter HIV-1 particles were produced by co-transfection of HEK 293T cells with pNL4.3-Env^−^-Luc and either an empty expression vector or an expression vector encoding either a parental or a recombinant *env*. For each individual recombinant variant, prior to their use for transfection, clones were verified by sequencing of the region encoding the recombinant gene as well as the vector-encoded promoter for its expression. Supernatants, containing virus stock, were harvested 48 h post transfection, and filtered trough a 0.45 µM filter. Production of viral particles was tested using an enzyme linked immunoassay for HIV-p24 antigen detection (Perkin Elmer, MA, USA) and 20 ng of p24 were used to infect 10^5^ 293T CD4^+^-CCR5^+^ cells in 24 wells plates. Forty-eight hours later, cells were washed twice in PBS and lysed in 25 mM Tris phosphate, pH 7.8, 8 mM MgCl2, 1 mM dithiothreitol, 1% triton X-100, and 7% glycerol for 10 min in a shaker at room temperature. The lysates were centrifuged and the supernatant was used to measure luciferase activity using a GloMax 96 Microplate Luminometer (Promega, WI, USA) following the instruction of the luciferase assay kit (Promega, WI, USA). For samples that yielded negative results in the luciferase assay, plasmids from at least three independent bacterial clones were tested.

### Recombination analysis of sequences sampled from nature

The HIV-1 group M envelope sequence alignment was retrieved from the Los Alamos National Laboratory (LANL) HIV Sequence Database (http://hiv-web.lanl.gov/). The alignment was reduced to subtype reference sequences (3 strains for each where available), 53 CRF strains (2 strains for each where available) and finally 197 apparently unique recombinants. Recombination was analyzed using the RDP [46], GENECONV [47], BOOTSCAN [48], MAXCHI [49], CHIMAERA [50], SISCAN [51], and 3SEQ [52] methods implemented in the program rdp3beta30 [53]. Default settings were used throughout except that: (1) only potential recombination events detected by four or more of the above methods, coupled with phylogenetic evidence of recombination were considered significant; (2) sequences were treated as linear; and (3) a window size of 30 variable nucleotide positions was used for the RDP method. Using the approach outlined in the rdp3 program manual (http://darwin.uvigo.es/rdp/rdp.html), the approximate breakpoint positions and recombinant sequence(s) inferred for every potential recombination event, were manually checked and adjusted where necessary using the phylogenetic and recombination signal analysis features available in rdp3. Breakpoint positions were classified as unknown if they were (1) detected at the 5′ and 3′ ends of the alignment but could have actually fallen outside the analysed region; or (2) within 20 variable nucleotide positions or 100 total nucleotides of another detected breakpoint within the same sequence (in such cases it could not be discounted that the actual breakpoint might not have simply been lost due to a more recent recombination event). All of the remaining breakpoint positions were manually checked and adjusted when necessary using mainly the MAXCHI and 3SEQ methods (using three sequence scans and the MAXCHI matrix method) but also the LARD matrix method (generated by the LARD two breakpoint scan; [54]), and the CHIMAERA method as tie breakers. The distribution of unambiguously detected breakpoint positions of all unique recombination events were analysed for evidence of recombination hot- and cold-spots with rdp3 as described by Heath *et al.* ([44]; a window size of either 50 or 200 nucleotides and 10 000 permutations). A normalised version of the breakpoint distribution plot described in that study was used in which the local probability values of breakpoint numbers (determined by a permutation test that takes into account that local degrees of sequence diversity influence the delectability of recombination events) were plotted instead of absolute breakpoint numbers.

### Schema analysis

PDB files detailing the three dimensional structures of both gp120 (PDB ID: 2B4C, determined by X-ray diffraction, resolution of 3.3 Å, 338 amino acids, [55]), and gp41 (PDB ID 1AIK, determined by X-ray diffraction, resolution of 2 Å, 70 amino acids, [56]) were obtained from http://www.rcsb.org. It is important to point out that these structures are partial and that we therefore only analysed a fraction of the structural interactions involved in Env folding. We performed SCHEMA predictions of recombination induced fold disruptions using the set of natural HIV *env* recombinants (described above) essentially as described in Lefeuvre et al. ([8]; See Protocol S1, Supplementary Analyses, for a description of the SCHEMA method). This involved: (1) computing protein fold disruption, or E, scores for each natural recombinant with identifiable parents; (2) based on every pair of parental sequences identified for the observed set of recombinants, simulating every possible recombinant that could have been produced with these parental sequence pairs that involved the exchange of the same number of non-synonymous polymorphisms as were exchanged during the actual recombination events; (3) calculating E scores for each of these simulated recombinants; and (4) using a permutation test to determine whether mean E scores calculated for the natural recombinants were significantly lower than mean E-scores for the same set of recombinants randomly drawn from the simulated recombinant datasets (Table S1). If fewer than 5% of simulated datasets had an average E score lower than that of the actual dataset (p<0.05) then this was taken to indicate that predicted fold disruptions incurred by real events were significantly less severe than if the observed distribution of breakpoints was uninfluenced by negative selection acting against recombinants with disrupted protein folding.

## Supporting Information

Protocol S1 Supplementary analyses. Schema analysis on the HIV envelope gene.
(0.02 MB DOC)

Table S1 Mutation and disruption value for gp41 and gp120 datasets real and simulated recombination events.
(0.04 MB DOC)
