# Supplementary material for: Molecular Mechanisms of Recombination Restriction in the Envelope Gene of the Human Immunodeficiency Virus
Source: PLoS Pathog. 2009 May 8;5(5):e1000418. doi: 10.1371/journal.ppat.1000418 (PMC2671596; doi:10.1371/journal.ppat.1000418)
Supplement: Protocol S1 — Supplementary analyses. Schema analysis on the HIV envelope gene. (0.02 MB DOC) [file ppat.1000418.s001.doc]

**Supplementary Analyses**

Schema analysis on the HIV envelope gene

Method

SCHEMA is a method designed by protein engineers to predict relative degrees of structural perturbation in recombinant proteins [3]. SCHEMA takes as input a PDB protein structure file and parental amino acid sequence files. It uses the protein structural information to properly fold the parental amino acid sequences and then identifies potentially interacting amino acid pairs based on their proximity (in this case within 4.5 Å) within the resulting folds. The amino acid contact map yielded by this process can then be used to determine the degree of fold disruption expected in any conceivable chimaera of the parental amino acid sequences. For all the amino acid residues that are potentially interacting within a folded chimaeric  protein, SCHEMA counts the number of instances where the interacting pairs are found in neither parent. Non-parental interacting amino acid pairs arise when the parental molecules differ from one another at two potentially interacting amino acid residues and the chimaera inherits one half of the potentially interacting pair from one parent and the other half from the other parent. Counts of these potentially non-interacting pairs in chimaeric proteins, called “E” values, have been shown to correlate directly with degrees of fold disruption experienced by the proteins. The value of E therefore corresponds with the expected degree of fold disruption.

Results

A first analysis performed over the four HIV subtype analysed in this study highlight the presence of disruption peaks in the middle of gp120 gene (data not shown). But because (i) analysing using SCHEMA a small number of chimera and (ii) in reason of the small length of sequence available with structural data, these analyses lack statistical basis and could only be used as a raw indication of how recombination cause protein misfolding. To circumvent this problem, we performed the same analysis over recombinant sequences available in public database and compare the disruption result to an exhaustive set of derived recombinant as described in reference 8. The results (Table S1), either for gp41 and gp120, clearly show the tendency for natural and selected recombinants (found in the database) to be less disruptive than if recombination occurred randomly. This implies the elimination from the population of viruses for which recombinant genes products are dysfunctional.
