# Supplementary material for: Molecular Mechanisms of Recombination Restriction in the Envelope Gene of the Human Immunodeficiency Virus
Source: PLoS Pathog. 2009 May 8;5(5):e1000418. doi: 10.1371/journal.ppat.1000418 (PMC2671596; doi:10.1371/journal.ppat.1000418)
Supplement: Table S1 — Mutation and disruption value for gp41 and gp120 datasets real and simulated recombination events. (0.04 MB DOC) [file ppat.1000418.s002.doc]

| **Table S1: Mutation and disruption value for gp41 and gp120 datasets real and simulated recombination events** | | | | | | | | | | | | |  |
| --- | --- | --- | --- | --- | --- | --- | --- | --- | --- | --- | --- | --- | --- |
|  |  |  |  | | | | | | | | | |  |
| **Genomic portion analysed** | | | **gp41** | | | **gp120** | | | | | | | |
| **Analysed position (aa) in relative to HXB2 gp160** | | | **546-581 / 628-661** | | | **84-130 / 195-492** | | | | | | | |
| **Recombination type** | | | **Real** | **Exhaustive genome** | **Exhaustive non synon.** | **Real** | | **Exhaustive genome** | **Exhaustive non synon.** | | | | |
|  |  |  |  |  |  |  | |  |  | | | | |
| **Monopartite** |  | **nb of event** | **8 (8^$^)** | **434** | **72** | **32 (32^$^)** | | **10178** | **2904** | | | | |
|  |  | **Mutation (m) mean ± sd (P-value*)** | **2.1 ± 1.9** | **3.2 ± 2.8 (0.13)** | **3.6 ± 0.7 (1.0)** | **16.2 ± 12.2** | | **20.5 ± 13.9 (6.6e-2)** | **17.2 ± 11.8 (1.0)** | | | | |
|  |  | **Disruption (E) mean ± sd (P-value*)** | **0.92 ± 1.4** | **1.4 ± 1.5 (0.046)** | **3.7 ± 3.6 (6.5e-3)** | **5.9 ± 5.7** | | **8.1 ± 10.9 (3.7e-2)** | **8.5 ± 5.3 (3.0e-4)** | | | | |
|  |  |  |  |  |  |  | |  |  | | | | |
| **^§^nucleotide position of the ORF portion analyzed relative to HXB2 sequence** | | | | | | | | | | | | | |
| **^$^number of event involving the transfer of non synonymous polymorphism** | | | | | | |  | | |  |  |  | |
| **^*^that real events are less mutative/disruptive than simulated events (exhaustive genome events and exhaustive non synonymous events). Considered significant when inferior to 0.05.** | | | | | | | | | | | | | |
